# pH sensors in the plant apoplast: a *sine qua non* of phenotypic plasticity

**DOI:** 10.3389/fpls.2023.1227279

**Published:** 2023-06-22

**Authors:** Isabel Cristina Vélez-Bermúdez, Wolfgang Schmidt

**Affiliations:** ^1^ Institute of Plant and Microbial Biology, Academia Sinica, Taipei, Taiwan; ^2^ Biotechnology Center, National Chung-Hsing University, Taichung, Taiwan; ^3^ Genome and Systems Biology Degree Program, College of Life Science, National Taiwan University, Taipei, Taiwan

**Keywords:** pH sensing, pH homeostasis, soil pH, abiotic stress, signal transduction, acclimation

## Introduction

Lacking the ability to escape from hostile environments, for plants, constant surveillance of their environment is pivotal for survival. As a key edaphic factor, the concentrations of protons—or pH—critically affects seedling establishment, growth, and fitness of the plant, leading to plant communities typical of the pH of the respective habitat (Ellenberg, 1958). Soil pH defines the activity of microbial communities and affects the composition of root exudates, which in turn define important ecological processes such as kin recognition, pathogen defense, and attraction of beneficial bacteria. Receiving information on external pH (pH_ext_) is critical to steer adaptive responses to a plethora of signals associated with the proton concentration in the rhizosphere. Surprisingly, plant pH sensing did not appear to be a popular concept during the past decades and was considered inessential as a ‘stand-alone’ system with the sole duty of monitoring the external proton concentration (Raven, 1990). However, more recently, the dogma that plants respond to rather than anticipate imbalances has been changed in favor to the latter view, a discourse that ultimately led to the discovery of systems that sense and signal pH_ext_.

## pH-dependent peptide signaling in the apoplast: sensing outside the box

In contrast to bacteria, mammals, and fungi, the mechanism(s) by which plants perceive information on pH_ext_ was a missing piece in the puzzle of their environmental surveillance, a gap that was filled only recently. *Arabidopsis* roots employ a bimodal pH-sensing system that calibrates the intricate tradeoff between growth and defense (Liu et al., 2022). At low pH, protonation of a sulfotyrosine in the root growth factor 1 (RGF1) peptide promotes binding to its receptor RGFR and the coreceptor SERK, forming a complex that supports growth of the root apical meristem. An increase in pH destabilizes the complex, stops meristem growth, and tips the balance towards a process that is prioritized under such conditions: pathogen defense. The presence of harmful microorganisms induces secretion of Pep (plant elicitor peptide), which binds to its receptor PEPR and the coreceptor BAK, limiting growth in favor of pattern-triggered immunity. At acidic pH, these interactions are inhibited by protonation of aspartic and glutamic acid residues in the Pep receptor (Liu et al., 2022).

A further pH-dependent regulatory system operating in *Arabidopsis* roots with a more specific task employs peptides from the CLAVATA3/Embryo Surrounding Region-Related (CLE) family. CLE45 was shown to suppress protophloem differentiation in the root apical meristem in an autocrine manner (i.e., by acting on the same cells that produce it) *via* pH-dependent binding to the receptor BAM3 (Rodriguez-Villalon, 2016; Diaz-Ardila et al., 2023). Conspicuously, both CLE45 and BAM3 are constitutively expressed in developing protophloem sieve elements (PPSEs), raising the questions as to how PPSEs defend themselves against autocrine activity of CLE45. Such self-inhibition is prevented by alkalization of the apoplast through inhibition of proton export, which desensitizes PPSE cell files against CLE45 signaling through deprotonation of the R4 residue of the peptide. The pH of neighboring cell files remains low, suppressing the PPSE cell fate in these cells by protonation of the pH-sensitive CLE45 residue. This mechanism safeguards proper cell patterning by regulating both apoplastic pH and CLE45 activity.

## Cellular ROS homeostasis defines alkalinity tolerance

These recent breakthroughs provide solid evidences for the ability of plants to sense pH_ext_, but also raise the question as to whether there is more to discover. Soil pH is associated with an intricate interplay of biotic and abiotic signals, making it tempting to suggest that plants employ mechanisms that orchestrate and prioritize the responses to such cues. In graminaceous crops, alkalinity was shown to negatively impact plant performance through an increase in H_2_O_2_ levels and subsequent oxidative cell damage. Genome-wide association studies revealed that in sorghum, alkalinity-induced accumulation of radical oxygen species (ROS) is caused by an atypical G protein γ subunit referred to as *Alkaline tolerance 1* (*AT1*), a gene that is homologous to *OsGS3*, *ZmGS3*, and *TaAT1* in rice, maize, and wheat, respectively (Zhang et al., 2023). AT1 regulates ROS levels *via* phosphorylation of the aquaporin PIP2. Inhibition of AT1 suppresses the phosphorylation and increases the activity of PIP2, thereby promoting the export of H_2_O_2_. While no regulatory control of this system has been reported (yet), pH-dependent regulation of AT1 activity would allow plants to sync ROS homeostasis with the prevailing pH conditions.

## Ambient pH reshapes transcriptomic and proteomic landscapes

Short-term changes in media pH cause pronounced alterations in gene expression and activity (Lager et al., 2010; Tsai and Schmidt, 2020; Chen et al., 2021; Tsai and Schmidt, 2021; Bailey et al., 2022), suggesting the employment of trans-acting elements that govern transcription and post-translational modifications in response to alterations in pH_ext_. Such changes were suggested to adapt the plant to constraints associated with the prevailing proton concentration in the rhizosphere, thereby optimizing tolerance to toxic ion species such as Al^3+^, modulating the acquisition of essential mineral nutrients, tuning cell wall extensibility, and recalibrating cellular pH homeostasis.

Inverse regulation of a large subset of genes in response to exposure to either acidic or alkaline conditions suggests putative roles in modulating the phenotypic readout of plants. While the function of most of the pH-responsive genes remains to be elucidated, in some cases the benefit of a strict regulation by pH_ext_ is obvious. pH-dependence of gene activity is particularly well-established for the case of iron uptake. Alkalinity restricts the solubility of iron by a factor of 1,000 for each unit increase in pH (Vélez-Bermúdez and Schmidt, 2023), requiring strategies to improve its uptake from recalcitrant pools. One such strategy is the secretion of the catecholic coumarins fraxetin (8-hydroxyscopoletin) and sideretin (5-hydroxyfraxetin), mobilizing iron by reducing ferric ions to the more soluble ferrous species and by chelating ferric iron, forming stable Fe(III)-coumarin complexes that may be taken up as such. Similar to the canonical FRO2/IRT1 iron uptake system, the production of coumarins is regulated by the transcription factor FIT, which also induces the expression of both the fraxetin-forming oxygenase *S8H* and the cytochrome P450 enzyme *CYP82C4*, which catalyzes the production of sideretin in the subsequent step in the biosynthetic pathway. Notably, alkaline conditions favor *S8H* expression and thus fraxetin production, an agent that is more efficient at high pH than its oxidized analog sideretin (Rajniak et al., 2018; Tsai et al., 2018). At alkaline pH, sideretin production is circumvented by suppression of *CYP82C4*, ensuring the secretion of the most effective compound under the respective conditions (Gautam et al., 2021). Also for this system, a pH-sensing and -signaling mechanism—although conspicuously required for this adaptation—has not yet been discovered.

## Nitrate-proton cotransport, the nouveau arrive in cellular pH homeostasis

The nitrate transporters NRT1.1 (CHL1) and NRT2.1 are mediating the uptake of nitrate from the soil solution. Both proteins import NO_3_^-^ ions together with protons and, thus, affect both apoplastic and cytosolic pH. Notably, NRT1.1 and NRT2.1 appear to be individually recruited at opposing pH_ext_ values, indicative of non-redundant, role of the two transporters.

How the expression and transport activities of the two nitrate transporters are coordinated with pH_ext_ is unclear at present. Induced expression of *NRT2.1* in response to alkaline conditions has been reported for different species and at different levels of gene expression, suggesting that increased NRT2.1 activity constitutes a robust and conserved response to high pH (Chen et al., 2021; Geng et al., 2021; Bailey et al., 2022; Jain and Schmidt, 2023). The role of this plasma membrane-bound transporter in the response to alkalinity appears to be univocal; it enriches the proton level in the cytosol. While a surplus in nitrate could be advantageous to support growth under alkaline condition, this does not seem to be the major goal here; in contrast to nitrate-treated plants, in which induction of *NRT2.1* is associated with increased abundance of transcripts encoding nitrogen-assimilating enzymes such as nitrate reductases (Vidal et al., 2013), high pH does not affect expression of the latter group of genes. In contrast to the wild type (and *nrt1.1* mutants), growth of *nrt2.1* seedlings at high pH was not associated with root growth cessation, suggesting that NRT2.1 plays a role in negatively regulating root growth (Jain and Schmidt, 2023). At low pH, *NRT1.1* is induced (while *NRT2.1* is repressed), possibly to modulate apoplastic pH under such conditions (Ye et al., 2021). Interestingly, it was reported that the expression of *NRT1.1* is increased by lowering the media pH both in the presence and absence of nitrate, suggesting that pH *per se* modulates *NRT1.1* transcript levels (Tsay et al., 1993). This observation can be considered as early evidence for the operation of a pH-sensing mechanism in *Arabidopsis* roots.

Assuming that pH_ext_ is chiefly communicated *via* peptide/receptor interactions, a nitrate-specific, peptide-mediated pH-sensing system that controls apoplastic and cytosolic proton homeostasis is a compelling scenario. Root nitrogen uptake is an integral of the shoot and root nitrogen status, which is communicated by small peptides of the C-terminally encoded peptide (CEP) family. CEPD1, CEPD2, and CEPDL2 are secondary signals derived from the action of CEP interacting with the shoot receptor kinase CEPR1 (Tabata et al., 2014) that systemically integrate the nitrogen demand of different plant parts. Interestingly, CEPDL2 specifically induces *NRT2.1* (but not *NRT1.1*; Ota et al., 2020), resembling high pH plants. In principle, the abundance of one or more CEPD1/2-CEPDL2 peptides could be coupled to a pH-sensing system, governing nitrate-anion cotransport to regulate cellular pH homeostasis under alkaline conditions.

## Leaves sense and communicate ambient pH

Reports on pH changes in the leaf apoplast are scarce. Transient alkalization has been observed in corn leaves upon exposure (of roots) to salinity stress, a process that was associated with abundance changes of leaf proteins involved in growth-relevant processes (Geilfus et al., 2017). Apoplast alkalization possibly derived from chloride-proton cotransport, depleting the H^+^ concentration outside the cell. Buffering apoplastic pH largely prevented the proteomic changes, suggesting that pH as such or associated downstream responses and not alterations in chloride concentration are causative for the changes.

The root is the only organ that is directly exposed to pH_ext_, suggesting that pH sensing is restricted to cells in contact to the soil solution. This appears, however, to be an invalid assumption. In sugar beet, exposure to different media pH altered the expression of proteins in both roots and leaves, robustly changing the abundance of several leaf proteins related to stress and defense (Geng et al., 2021). Similarly, in *Arabidopsis* the expression of a relatively large subset of proteins was altered upon short-term exposure to acidic or alkaline media pH (Jain and Schmidt, 2023), suggesting that information on pH_ext_ is communicated from roots to shoots. How such information migrates systemically is unclear. Plausible candidates mediating long-distance pH signaling are Ca^2+^ ions. Alterations in cytosolic pH were shown to be associated with Ca^2+^ transients (Behera et al., 2018), with the latter being able to migrate within the plant (Tian et al., 2020). Thus, information on proton concentration in the rhizosphere is possibly conveyed to above-ground organs in the ‘currency’ of Ca^2+^ ions. Together, these data imply that pH_ext_ sensing is not restricted to roots but occurs also in leaves, tuning gene expression and essential processes such as leaf expansion and meristem activity to the concurrent edaphic conditions. It thus appears that the supposition that leaves can sense the pH in the apoplast is well-supported by experimental data; however, direct evidence for the operation of a pH-sensing system is still missing.

## pH_ext_-sensing systems: a can of worms?

Phenotypical plasticity, the ability to produce different phenotypes in response to environmental cues, is essential to compensate for the lack of behavioral resources. The pH of the soil solution defines the edaphic conditions the plant is exposed to, triggering responses that govern the allocation of resources and the prioritization of adaptive measures to modulate growth, defense, and development. Hence, pH_ext_ can be considered as a major player of phenotypic plasticity, setting the stage for plant performance and fitness. While we can only speculate when it comes to the question as to how many pH-sensing systems plants have evolved, it seems fair to state that a one-for-all mechanism that perceives pH_ext_ and dictates all downstream responses associated with the prevailing conditions is an unlikely scenario. It is further conceivable to assume that the specificity of the cue and the required response necessitates perception cascades connected to or intertwined with other signaling pathways, mechanisms that are adopted to secure the myriad of functional readouts that plants display upon perception of the large combination of biotic and abiotic cues. Thus, it appears that the puzzle of peptide- or transceptor-based perception of environmental signals in general and the sensing of proton concentrations in the apoplast in particular is far from being complete. It thus seems safe to assume that the inventory of pH-sensing systems in plants is likely to be extended soon, outdating this article in due course.

## Author contributions

IV-B and WS performed the literature search and wrote the manuscript. WS suggested the concept of the review. Both authors approved the submitted version. All authors contributed to the article and approved the submitted version.
